# Integrated Analysis for Identifying Radix Astragali and Its Adulterants Based on DNA Barcoding

**DOI:** 10.1155/2014/843923

**Published:** 2014-08-27

**Authors:** Sihao Zheng, Dewang Liu, Weiguang Ren, Juan Fu, Linfang Huang, Shilin Chen

**Affiliations:** ^1^Institute of Medicinal Plant Development, Chinese Academy of Medical Sciences, Peking Union Medical College, Beijing 100193, China; ^2^School of Pharmacy, Inner Mongolia Medical University, Inner Mongolia 010080, China; ^3^Institute of Chinese Materia Medica, China Academy of Chinese Medical Sciences, Beijing 100700, China

## Abstract

Radix Astragali is a popular herb used in traditional Chinese medicine for its proimmune and antidiabetic properties. However, methods are needed to help distinguish Radix Astragali from its varied adulterants. DNA barcoding is a widely applicable molecular method used to identify medicinal plants. Yet, its use has been hampered by genetic distance, base variation, and limitations of the bio-NJ tree. Herein, we report the validation of an integrated analysis method for plant species identification using DNA barcoding that focuses on genetic distance, identification efficiency, inter- and intraspecific variation, and barcoding gap. We collected 478 sequences from six candidate DNA barcodes (ITS2, ITS, *psb*A-*trn*H, *rbc*L, *mat*K, and COI) from 29 species of Radix Astragali and adulterants. The internal transcribed spacer (ITS) sequence was demonstrated as the optimal barcode for identifying Radix Astragali and its adulterants. This new analysis method is helpful in identifying Radix Astragali and expedites the utilization and data mining of DNA barcoding.

## 1. Introduction 

Radix Astragali (Huang Qi), a commonly used Chinese medicinal material, is mainly sourced from the plants of* Astragalus membranaceus* and* Astragalus mongholicus *according to Chinese Pharmacopoeia (2010 edition). Radix Astragali is widely used for its antiperspirant, antidiuretic, and antidiabetic properties and as a tonic drug [1–3]. It possesses various beneficial compounds, including astragalosides, isoflavonoids, isoflavones, isoflavan, and pterocarpan glycosides [4–6].

Due to the high market demand for Radix Astragali, a diverse group of adulterants with similar-morphological characteristics from genuses, such as* Astragalus*,* Hedysarum*, and* Malva* are often used in its stead [7]. The traditional methods used to identify Radix Astragali for use as a medicinal material, such as morphological and microscopic identification [8], thin-layer chromatography and Ultraviolet spectroscopy [9], Fourier Transform infrared spectroscopy (FTIR) [10], and high performance liquid chromatography (HPLC) [11], all, require specialized equipment and training. Several PCR-based molecular methods have been developed, providing an alternative means of identification. Multiplex PCR methods of DNA fragment analysis, such as randomly amplified polymorphic DNA (RAPD) [12] or amplified fragment length polymorphism (AFLP) [13], are unstable for the results to identify. DNA barcoding is a widely used molecular marker technology, first proposed by Hebert et al. [14, 15]. It uses a standardized and conserved, but diverse, DNA sequence to identify species and uncover biological diversity [16, 17]. In previous studies, various coding sequences for identifying Radix Astragali and its adulterants have been used, such as the 5S-rRNA spacer domain [18], 3′ untranslated region (3′ UTR) [19], ITS (internal transcribed spacer region) and 18S rRNA [3, 20, 21], ITS2 [22], ITS1 [6],* mat*K (maturase K) and* rbc*L (ribulose 1, 5-bisphosphate carboxylase) of chloroplast genome, and coxI (cytochrome c oxidase 1) of the mitochondrial genome [23]. However, sequence analysis was mainly focused on genetic distance, variable sites, amplified polymorphisms, and the use of a modified neighbor-joining (NJ) algorithm, Bio-NJ tree, which were basic analyses limited to particular species. A more effective method of molecular identification is necessary. The current study evaluates the identification reliability and efficiency of DNA barcoding for the identification of Radix Astragali using six indicators of genetic distance, identification efficiency, intra- and interspecific variation, gap rate, and barcoding gap. Six barcodes were selected for identification because they are commonly used in plant, especially in medicinal plant. We collected Radix Astragaliand several of its adulterants reported in previous research and downloaded the genetic sequences from the GenBank database. A total of 29 species (including 19 species of* Astragalus*) and 478 sequences from six barcodes were used to validate the new method for identifying Radix Astragali and adulterants and to accelerate the data utilization of DNA barcoding.

## 2. Materials and Methods

### 2.1. Materials Information

A total of 77 specimens were collected from two origins of Radix Astragali, along with seven adulterants. Radix Astragali specimens were collected from Inner Mongolia, Shaan xi, and Gan su provinces in the People's Republic of China, which are the main producing areas. The collection information is shown in Table 1. All corresponding voucher specimens were deposited in the Herbarium of the Institute of Medicinal Plant Development at the Chinese Academy of Medical Sciences in Beijing, China. The GenBank accession number of the ITS2 in this experiment was orderly KJ999296–KJ999344, the accession number of ITS sequences was orderly KJ999345–KJ999416, and the accession number of* psb*A-*trn*H was orderly KJ999256–KJ999295. The sequences added in the subsequent analysis, including ITS, ITS2,* psb*A-*trn*H,* mat*K, and* rbc*L, were downloaded from the GenBank database.

### 2.2. DNA Extraction, PCR Amplification, and Sequencing

The material specimens were naturally dried and 30 mg of dried plant material was used for the DNA extraction. Samples were rubbed for two minutes at a frequency of 30 r/s in a FastPrep bead mill (Retsch MM400, Germany), and total genomic DNA was isolated from the crushed material according to the manufacturer's instructions (Plant Genomic DNA Kit, Tiangen Biotech Co., China). We made the following modifications to the protocol: chloroform was diluted with isoamyl alcohol (24 : 1 in the same volume) and buffer solution GP2 with isopropanol (same volume). The powder, 700 *μ*L of 65°C GP1, and 1 *μ*L *β*-mercaptoethanol were mixed for 10–20 s before being incubated for 60 minutes at 65°C. Then, 700 *μ*L of the chloroform:isoamyl alcohol mixture was added and the solution was centrifuged for 5 minutes at 12000 rpm (~13400 ×g). Supernatant was removed and placed into a new tube before adding 700 *μ*L isopropanol and blending for 15–20 minutes. The mixture was centrifuged in CB3 spin columns for 40 s at 12000 rpm. The filtrate was discarded and 500 *μ*L GD (adding quantitative anhydrous ethanol before use) was added before centrifuging at 12000 rpm for 40 s. The filtrate was discarded and 700 *μ*L PW (adding quantitative anhydrous ethanol before use) was used to wash the membrane before centrifuging for 40 s at 12000 rpm. This step was repeated with 500 *μ*L PW, followed by a final centrifuge for 2 minutes at 12000 rpm to remove residual wash buffer. The spin column was dried at room temperature for 3–5 minutes and then centrifuged for 2 minutes at 12000 rpm to obtain the total DNA.

General PCR reaction conditions and universal DNA barcode primers were used for the ITS, ITS2, and* psb*A-*trn*H barcodes, as presented in Table 2 [24–26]. PCR amplification was performed on 25-*μ*L reaction mixtures containing 2 *μ*L DNA template (20–100 ng), 8.5 *μ*L ddH2O, 12.5 *μ*L 2× Taq PCR Master Mix (Beijing TransGen Biotech Co., China), and 1/1-*μ*L forward/reverse (F/R) primers (2.5 *μ*M). The reaction mixtures were amplified in a 9700 GeneAmp PCR system (Applied Biosystems, USA). Amplicons were visualized by electrophoresis on 1% agarose gels. Purified PCR products were sequenced in both directions using the ABI 3730XL sequencer (Applied Biosystems, USA).

### 2.3. Sequence Assembly, Alignment, and Analysis

Sequencing peak diagrams were obtained and proofread, and then contigs were assembled using a CodonCode Aligner 5.0.1 (CodonCode Co., USA). Complete ITS2 sequences were obtained using the HMMer annotation method, based on the Hidden Markov model (HMM) [27]. All of the sequences were aligned using ClustalW, in combination with 317 sequences from six commonly used barcodes (ITS2, ITS,* psb*A-*trn*H,* mat*K,* rbc*L, and COI), which were downloaded from the GenBank database (Table 3). Sequence genetic distance and GC content were calculated using the maximum composite likelihood model. Maximum likelihood (ML) trees were constructed based on the Tamura-Nei model, and bootstrap tests were conducted using 1000 repeats to assess the confidence of the phylogenetic relationships by MEGA 6.0 software [28]. The barcoding gap, defined as the spacer region between intra- and interspecific genetic variations, and identification efficiency, based on BLAST1 and K2P nearest distance, were performed by the Perl language algorithm (Putty) [25, 29, 30].

## 3. Results

### 3.1. Sequence Information and Identification Efficiency

A total of 478 sequences for six barcodes were analyzed, from which 161 sequences were obtained from Astragalus Radix and its adulterants. Sequence information and identification success rates are listed in Table 4. The average GC content of six barcodes was discrepant, and ITS and ITS2 regions from nuclear ribosomal DNA performed higher than other barcodes (52.97% versus 50.80%). Among the six barcodes, ITS2 provided the largest average genetic distance (1.0792), and rbcL was the smallest (0.0349). All of the six barcodes obtained a zero value for the minimum genetic distance. In terms of identification efficiency, the nearest distance method was superior to the BLAST1 method for all of the six barcodes. Moreover, ITS and the* psb*A-*trn*H and* mat*K regions provided a higher rate of success than the other three barcodes using the BLAST1 method. However,* mat*K, ITS, and* psb*A-*trn*H performed better than the other three barcodes, based on the nearest distance method. ITS and* psb*A-*trn*H obtained higher genetic distances, so the* mat*K, ITS, and* psb*A-*trn*H barcodes were the preferable methods for identifying Radix Astragali and its adulterants based on superior sequencing efficiency and identification efficiency.

### 3.2. Intra- and Interspecific Variation Analysis Using Six Parameters

Six parameters to analyze intraspecific variation and interspecific divergence were employed to assess the utility of six DNA barcodes (Table 5). We expected the “minimum interspecific distance” would be higher than the “coalescent depth” (maximum intraspecific distance). Therefore, we first utilized the “gap rate” to indicate the distinctness, calculated by the formula: (minimum interspecific distance − maximum intraspecific distance)/minimum interspecific distance. Results show that the ITS2, COI,* mat*K, and* rbc*L regions outperformed the ITS and* psb*A-*trn*H regions for gap rates. However, when we compared all of the average inter- and intraspecific distances, the ITS2,* rbc*L,* mat*K, and* psb*A-*trn*H regions performed better than the ITS and COI regions. Therefore, in terms of intra- and interspecific variation, ITS2,* mat*K, and* rbc*L are the preferable options for identifying Radix Astragali and its adulterants.

### 3.3. Barcoding Gap Analysis

**Figure 1:**
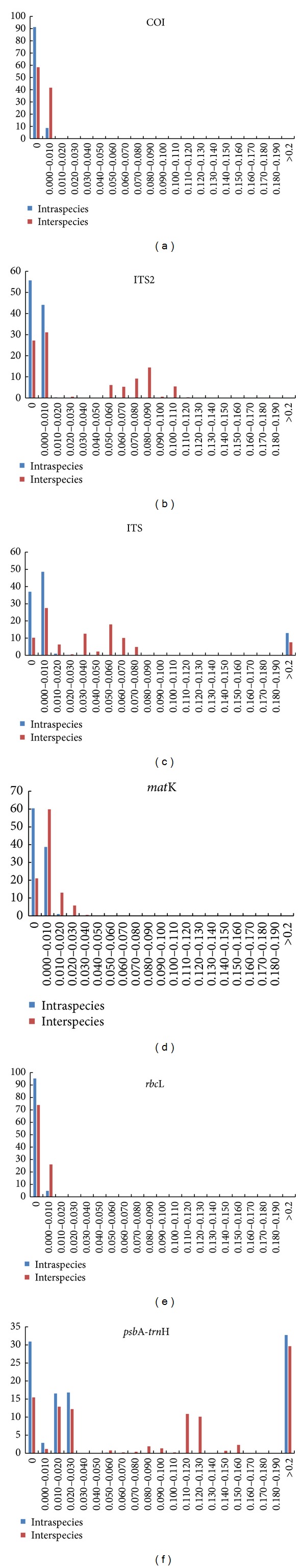
Barcoding gap for six barcodes.

Analysis of the DNA barcoding gap presents the divergence of inter- and intraspecies and indicates separate, nonoverlapping distribution between specimens in an ideal situation [25]. In our study (Figure 1), the* rbc*L, COI, ITS, and* mat*K regions possessed less relative distribution of inter- and intraspecific variation than* psb*A-*trn*H and ITS2, although there were no nonoverlapping regions for the six barcodes. Hence, the* rbc*L, COI, ITS, and* mat*K regions are more successful at identifying Radix Astragali and its adulterants, from the standpoint of barcoding gap analysis.

### 3.4. ML Tree Analysis

**Figure 2:**
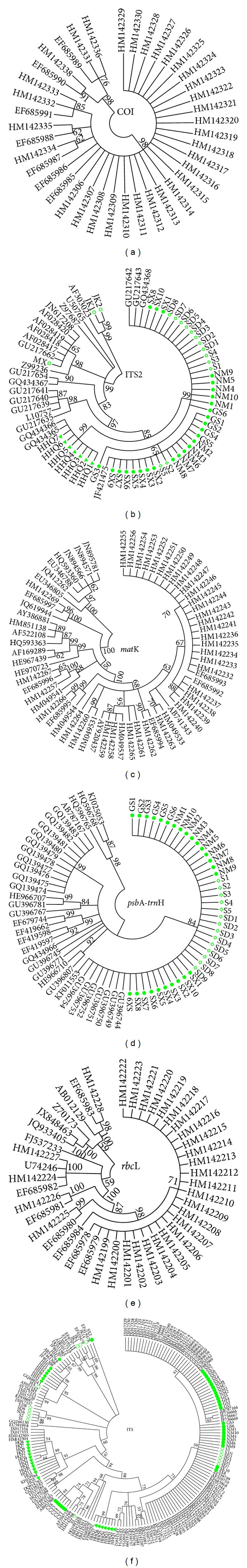
ML tree for six barcodes. ∗The different color and shape for different species in clusters presented the identification of different barcodes.

Maximum likelihood (ML) is a general statistical criterion in widespread use for the inference of molecular phylogenies [31]. An ML tree visually revealed the relationship between species. As the results show (Figure 2),* psb*A-*trn*H successfully differentiated Radix Astragali and its adulterants. Furthermore, it produced areas of obvious separation for Radix Astragali. The remaining five barcodes also differentiated Radix Astragali and its adulterants. Each species clustered together, separate from other species. Considering the difficult amplification and sequencing and fast and accurate identification purpose of DNA barcoding, we did not add all the sequence data of ITS2 and* psb*A-*trn*H to build ML tree and subsequent analysis.

## 4. Discussion and Conclusions

Radix Astragali is reported to possess 47 bioactive compounds and has many bioactive properties [32–37]. Various Radix Astragali preparations are commercially available, not only in China as a TCM component, but also in the United States, as dietary supplements [38]. However, due to increasing demand, substitutes and adulterants have flooded the market. Traditional identification methods, such as morphological and microscopic methods, are limited by the lack of explicit criteria for character selection or coding and, thus, mainly depend on subjective assessments. Although chemical methods are able to distinguish between different species, it is difficult to differentiate sibling species that possess similar chemical compositions. In addition, chemical methods are unable to provide accurate species authentication. Several types of molecular markers for characterizing genotypes are useful in identifying plant species. For example, RAPD has been used to estimate genetic diversity in plant populations based on amplification of random DNA fragments and comparisons of common polymorphisms. DNA barcoding is advocated for species identification, due to its universal applicability, simplicity, and scientific accuracy. However, the analysis methods for DNA barcodes were limited. With the development of molecular biology and bioinformatics, a more improved analytic method for DNA barcoding can be established to identify Radix Astragali and closely related species.

In this study, we validated a new analytical method for identifying Radix Astragali using DNA barcoding. Seventy-seven specimens of Radix Astragali and its adulterants were collected, and the sequences of 29 species reported in the literature were downloaded from the GenBank database. Based on the 478 sequences for six barcodes (ITS2, ITS from nuclear genome;* psb*A-*trn*H,* rbc*L, and* mat*K from chloroplast genome; COI from mitochondrial genome), genetic distance and ML Tree were calculated by MEGA 6.0 software, and identification efficiency, intra- and interspecific variation, and barcoding gap were calculated using the Perl language algorithm. Results of the six indicators assessed are shown in Table 6. ITS and* psb*A-*trn*H outperformed other barcodes in terms of identification efficiency. ITS2 performed better in terms of genetic distance, gap rate, and inter- and intraspecific variation.* Rbc*L performed better in terms of barcoding gap and inter- and intraspecific variation. Although ITS2 was part of the ITS sequence, it performed poorly in identification efficiency. Therefore, we suggest that the ITS sequence is the optimal barcode, and that the* psb*A-*trn*H region is a complementary barcode for identifying Radix Astragali and its adulterants.

In conclusion, we describe a new analytical method for the use of DNA barcoding in the identification of Radix Astragali. Six indicators, including average genetic distance, BLAST1 and the nearest distance method for identification efficiency, inter- and intraspecific variation, and gap rate were tested to evaluate six DNA barcodes using bioinformatics software and the Perl language algorithm. The ITS sequence was the optimal barcode for identifying Radix Astragali and its adulterants. This method provides a novel means for accurate identification of Radix Astragali and its adulterants and improves the utilization of DNA barcoding in identifying medicinal plant species.

## Figures and Tables

**Table 1:** Taxon sampling information of astragalus and its adulterants.

Experiment number	species	Sampling spot	
S1-S5	*Astragalus membranaceus *	Shaanxi	China
SD1-SD9	*Astragalus membranaceus *	Shaanxi	China
GS1-GS6	*Astragalus mongholicus *	Gansu	China
NM1-NM10	*Astragalus mongholicus *	Neimeng	China
SX1-SX10	*Astragalus mongholicus *	Shanxi	China
HHQ1-HHQ7	*Astragalus chinensis *	Beijing	China
CY1-CY6	*Astragalus scaberrimus *	Beijing	China
JK1-JK3	*Malva pusilla *	Shaanxi	China
MX	*Medicago sativa *	Shaanxi	China
HH1-HH7	*Melilotus officinalis *	Shaanxi	China
HQ1-HQ12	*Hedysarum polybotrys *	Gansu	China
XJ	*Astragalus adsurgens *	Beijing	China

**Table 2:** Primers and PCR reaction conditions.

Primer name	Primer sequences (5′-3′)	PCR reaction condition
ITS2		
2F	ATGCGATACTTGGTGTGAAT	94°C 5 min;
3R	GACGCTTCTCCAGACTACAAT	94°C 30 s, 56°C 30 s, 72°C 45 s, 40 cycles; 72°C 10 min;
ITS		
4R	TCCTCCGCTTATTGATATGC	94°C 5 min;
5F	GGAAGTAAAAGTCGTAACAAGG	94°C 1 min, 50°C 1 min, 72°C 1.5 min + 3 s/cycle, 30 cycles; 72°C 7 min;
*psb*A		
fwdPA	GTTATGCATGAACGTAATGCTC	94°C 4 min;
*trn*H		
rev TH	CGCGCATGGTGGATTCACAATCC	94°C 30 s, 55°C 1 min, 72°C 1 min, 35 cycles; 72°C 10 min;

**Table 3:** Sequences from GenBank for identifying *Astragalus* and its adulterants.

Region	Family	Species	Accession number
ITS2	Fabaceae	*Melilotus officinalis *	U50765, Z97687
	Fabaceae	*Astragalus adsurgens *	L10757, GU217639, GU217640, GU217641
	Fabaceae	*Astragalus chinensis *	GQ434365, GQ434366
	Fabaceae	*Hedysarum polybotrys *	GQ434367
	Fabaceae	*Astragalus mongholicus *	GQ434368, GU217643
	Fabaceae	*Astragalus mongholicus var. dahuricus *	GU217635
	Fabaceae	*Astragalus membranaceus *	GU217642, JF421475
	Fabaceae	*Caragana sinica *	GU217654
	Fabaceae	*Medicago sativa *	GU217662, Z99236, AF028417, JN617208
	Fabaceae	*Medicago sativa subsp. caerulea *	AF028418
	Fabaceae	*Medicago sativa subsp. glomerata *	AF028419
	Fabaceae	*Medicago falcata *	AF028420
	Malvaceae	*Alcea rosea *	AF303023

ITS	Fabaceae	*Astragalus membranaceus *	AF359749, EF685968, EU852042, FJ572044, GU289659
			GU289660, GU289661, GU289662, GU289663, GU289664
			HM142272, HM142273, HM142274, HM142275, HM142276
			HM142277, HM142278, HM142279, HM142280, HM142281
			HQ891827, JX017320, JX017321, JX017322, JX017323
			JX017324, JX017325, JX017326, JX017327, JX017328
			JX017329, JX017330, JX017331, JX017332, AF121675
	Fabaceae	*Astragalus mongholicus *	AF359750, EF685969, HM142282, HM142283, HM142284
			HM142285, HM142286, HM142287, HM142288, HM142289
			HM142290, JF736665, JF736666, JF736667, JF736668
			JF736669, AB787166
	Fabaceae	*Astragalus propinquus *	AF359751
	Fabaceae	*Astragalus lepsensis *	AF359752
	Fabaceae	*Astragalus aksuensis *	AF359753, AB231091
	Fabaceae	*Astragalus hoantchy *	AF359754, AF521952
	Fabaceae	*Astragalus hoantchy subsp. dshimensis *	AF359755
	Fabaceae	*Astragalus lehmannianus *	AF359756
	Fabaceae	*Astragalus sieversianus *	AF359757
	Fabaceae	*Astragalus austrosibiricus *	AF359758
	Fabaceae	*Astragalus uliginosus *	EF685970
	Fabaceae	*Astragalus scaberrimus *	AB051988
	Fabaceae	*Astragalus chinensis *	FJ980292, HM142297, AF121681
	Fabaceae	*Astragalus borealimongolicus *	HM142291, HM142292, HM142293, HM142294, HM142295
			HM142296
	Fabaceae	*Astragalus adsurgens *	HM142298, HM142299, HQ199326
	Fabaceae	*Astragalus mongholicus var. dahuricus *	HM142300, KC262199
	Fabaceae	*Astragalus zacharensis *	HM142301
	Fabaceae	*Astragalus melilotoides *	HM142302
	Fabaceae	*Astragalus scaberrimus *	HM142303
	Fabaceae	*Astragalus sieversianus *	AB741299
	Fabaceae	*Oxytropis anertii *	EF685971
	Fabaceae	*Caragana sinica *	DQ914785, FJ537284, GQ338283
	Fabaceae	*Glycyrrhiza pallidiflora *	EU591998, GQ246130
	Fabaceae	*Melilotus officinalis *	AB546796, JF461307, JF461308, JF461309, DQ311985
	Fabaceae	*Medicago sativa *	GQ488541, AF053142, AY256392, JX017335, JX017336
			JX017337, KF938697
	Fabaceae	*Oxytropis caerulea *	GU217599, HQ199316
	Fabaceae	*Hedysarum vicioides *	HM142304, HM142305
	Fabaceae	*Hedysarum polybotrys *	JX017333, JX017334, KF032294
	Malvaceae	*Malva neglecta *	EF419478, EF419479
	Malvaceae	*Alcea rosea *	AH010172, EF419544, EF679714, JX017319

*psb*A-*trn*H	Fabaceae	*Astragalus membranaceus f. pallidipurpureus *	GQ139474
	Fabaceae	*Astragalus adsurgens *	GU396749, GU396750, GU396751, KF011553
	Fabaceae	*Astragalus mongholicus *	GU396754, AB787167
	Fabaceae	*Astragalus membranaceus *	GQ139475, GQ139476, GQ139477, GQ139478, GQ139479
			GQ139480, GQ139481, GQ139482, GQ139483, GU396752
			GU396753
	Fabaceae	*Caragana sinica *	GU396767, KJ025053
	Fabaceae	*Oxytropis caerulea *	GU396771
	Fabaceae	*Medicago sativa *	GU396781, HQ596768, HE966707
	Fabaceae	*Glycyrrhiza pallidiflora *	GU396807
	Fabaceae	*Melilotus officinalis *	HE966710
	Malvaceae	*Malva neglecta *	EF419597, EF419598, HQ596765, HQ596765
	Malvaceae	*Alcea rosea *	EF419662, EF679744

*mat*K	Fabaceae	*Astragalus membranaceus *	EF685992, HM142232, HM142233, HM142234, HM142235
			HM142236, HM142237, HM142238, HM142239, HM142240
			HM142254
	Fabaceae	*Astragalus mongholicus *	EF685993, HM142241, HM142242, HM142243, HM142244
			HM142245, HM142246, HM142247, HM142255, HM142256
	Fabaceae	*Astragalus uliginosus *	EF685994, HM142262
	Fabaceae	*Astragalus mongholicus var. dahuricus *	HM049531, HM142260
	Fabaceae	*Astragalus chinensis *	HM049533, HM142263
	Fabaceae	*Astragalus adsurgens *	HM049537, HM142258, HM142259, AY920437
	Fabaceae	*Astragalus borealimongolicus *	HM142248, HM142249, HM142250, HM142251, HM142252
			HM142253
	Fabaceae	*Astragalus zacharensis *	HM142261
	Fabaceae	*Astragalus melilotoides *	HM142264
	Fabaceae	*Astragalus scaberrimus *	HM142265
	Fabaceae	*Astragalus sieversianus *	AB741343
	Fabaceae	*Medicago sativa *	AF522108, HQ593363, HM851138, AY386881, HE967439
			AF169289
	Fabaceae	*Oxytropis anertii *	EF685995, HM142266
	Fabaceae	*Oxytropis caerulea *	HM049544
	Fabaceae	*Glycyrrhiza pallidiflora *	EF685997, HM142269, JQ619944
	Fabaceae	*Hedysarum vicioides *	EF685996, HM142257, HM142267
	Fabaceae	*Caragana sinica *	HM049541
	Fabaceae	*Melilotus officinalis *	HE970723
	Malvaceae	*Malva neglecta *	EU346788, HQ593360, JN894566, JN894571, JN895781
			JQ412262,
	Malvaceae	*Alcea rosea *	EU346805

*rbc*L	Fabaceae	*Medicago sativa *	Z70173
	Fabaceae	*Astragalus membranaceus *	EF685978, HM142199, HM142200, HM142201, HM142202
			HM142203, HM142204, HM142205, HM142206, HM142207
			HM142221
	Fabaceae	*Astragalus mongholicus *	EF685979, HM142208, HM142209, HM142210, HM142211
			HM142212, HM142213, HM142214, HM142222, HM142223
	Fabaceae	*Astragalus uliginosus *	EF685980, HM142225
	Fabaceae	*Hedysarum vicioides *	EF685982, U74246, HM142224, HM142227,
	Fabaceae	*Astragalus adsurgens *	EF685984
	Fabaceae	*Astragalus borealimongolicus *	HM142215, HM142216, HM142217, HM142218, HM142219
			HM142220,
	Fabaceae	*Oxytropis anertii *	EF685981, HM142226
	Fabaceae	*Glycyrrhiza pallidiflora *	EF685983, AB012129, HM142228
	Fabaceae	*Caragana sinica *	FJ537233
	Fabaceae	*Melilotus officinalis *	JQ933405, JX848463

**Table 4:** The information of identification efficiency for six barcodes.

Markers	COI	ITS2	ITS	*mat*K	*rbc*L	*psb*A-*trn*H
Number of sequences	39	72	185	65	43	74
Average GC content/%	43.29	50.80	52.97	31.14	42.88	21.77
Genetic distance						
Min	0.0000	0.0000	0.0000	0.0000	0.0000	0.0000
Max	0.0086	7.9494	5.3130	0.2801	0.0349	2.2701
Average	0.0019	1.0792	0.3508	0.0711	0.0116	0.5080
Identification efficiency/%						
BLAST 1/%	10.26	12.50	30.81	29.23	23.26	29.73
Nearest distance/%	33.33	27.78	52.43	66.15	37.21	41.89

**Table 5:** Analysis of interspecific divergence and intraspecific variation for six barcodes.

Marker (Mean ± SD)	COI	ITS2	ITS	*mat*K	*rbc*L	*psb*A-*trn*H
Theta	2.2260 ± 6.2961	0.0030 ± 0.0046	0.0271 ± 0.0404	0.0021 ± 0.0035	0.0011 ± 0.0020	0.2415 ± 0.4777
Coalescent depth	0.0001 ± 0.0004	0.0040 ± 0.0046	0.1423 ± 0.3958	0.0032 ± 0.0050	0.0016 ± 0.0030	0.4109 ± 0.5683
All intraspecific distance	9.3280 ± 0.0003	0.0021 ± 0.0024	0.1153 ± 0.3051	0.0014 ± 0.0022	0.0002 ± 0.0011	0.3093 ± 0.4300
Theta prime	0.0012 ± 0.0008	0.0617 ± 0.0302	0.0603 ± 0.0371	0.0091 ± 0.0061	0.0024 ± 0.0035	0.3083 ± 0.2887
Minimum interspecific distance	0.0008 ± 0.0010	0.0440 ± 0.0386	0.0168 ± 0.0196	0.0066 ± 0.0066	0.0023 ± 0.0035	0.0423 ± 0.0380
All interspecific distance	0.0007 ± 0.0010	0.0343 ± 0.0389	0.1066 ± 0.2833	0.0071 ± 0.0064	0.0015 ± 0.0029	0.3166 ± 0.4070
Gap rate/%	87.50	90.91	/	51.52	30.43	/

**Table 6:** Six indicators assessed for DNA barcoding.

DNA barcodes	Parameters						
	Average genetic distance	Identification efficiency		Gap rate	Inter- to intraspecific variation	Barcoding gap	Total score
		BLAST1	Nearest distances				
ITS2	8	12	8	8	8	4	48
ITS	6	28	22	0	0	6	62
*psb*A-*trn*H	6	26	18	0	2	2	54
*rbc*L	4	12	14	4	6	8	48
*mat*K	4	14	24	4	4	2	52
COI	2	6	10	6	0	6	30

∗The total score of six parameters was set by 10, 30, 30, 10, 10, and 10 in order. Identification efficiency based on two methods was set by 30 score because of its importance for identification.
